# Societal drivers of human echinococcosis in China

**DOI:** 10.1186/s13071-022-05480-8

**Published:** 2022-10-22

**Authors:** Li-Ying Wang, Min Qin, Laurent Gavotte, Wei-Ping Wu, Xixi Cheng, Jia-Xi Lei, Jun Yan, Roger Frutos

**Affiliations:** 1 Chinese Centre for Disease Control and Prevention (Chinese Centre for Tropical Diseases Research), Key Laboratory of Parasite and Vector Biology, National Institute of Parasitic DiseasesWHO Collaborating Centre for Tropical DiseasesNational Centre for International Research On Tropical Diseases, Shanghai, People’s Republic of China; 2 NHC Key Laboratory of Echinococcosis Prevention and Control, Tibet Center for Disease Control and Prevention, Lhasa, China; 3 Espace-Dev, UMR 228, Université de Montpellier, Montpellier, France; 4 Cirad, UMR 17, Intertryp, Campus international de Baillarguet, Montpellier, France; 5 Chinese Centre for Disease Control and Prevention, Beijing, China

**Keywords:** Echinococcosis, Susceptibility factors, Cluster analysis, Qinghai-Tibet Plateau, Non-Qinghai-Tibet Plateau

## Abstract

**Background:**

Echinococcosis is a parasitic zoonotic disease that threatens human health and economic development. In China, 370 counties are endemic for echinococcosis. Qinghai-Tibet Plateau has the most patients and people at risk. Therefore, analyzing the societal factors related to susceptibility to the disease is critical for efficient prevention and control of echinococcosis.

**Methods:**

The demographic characteristics and lifestyle of echinococcosis cases were clustered using K-means cluster analysis to determine the main factors of risk of echinococcosis.

**Results:**

Middle-aged and young people as well as those with a low education level and herdsmen are at risk of contracting echinococcosis. Nomadism, domestic and feral dogs in the surrounding environment, and drinking heavily polluted natural surface water are the main behavioral risk factors. The cystic echinococcosis (CE) and alveolar echinococcosis (AE) cluster analysis focused on female, middle-aged, and young people, winter settlement and summer nomadism, and domestic and feral dogs in the surrounding environment. There were significant differences in lifestyle between Qinghai-Tibet Plateau cases and non-Qinghai-Tibet-Plateau cases.

**Conclusion:**

According to the distribution of cases and CE and AE, this study identified the factors of risk of echinococcosis in the Qinghai-Tibet Plateau and non-Qinghai-Tibet Plateau. Adapted control techniques appropriate for the various epidemic areas should be established to serve as a reference for echinococcosis prevention.

**Graphical Abstract:**

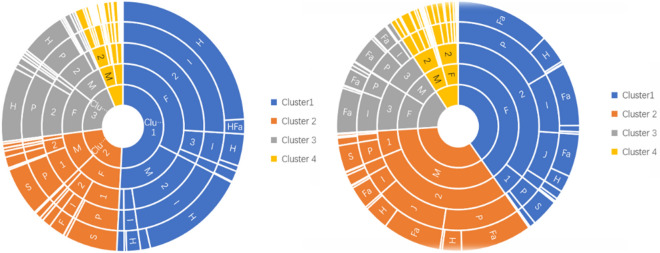

## Introduction

Echinococcosis is a zoonotic parasitic disease caused by the larval stages of cestodes of *Echinococcus* found in humans and animals. China is one of the countries with the most serious epidemics of echinococcosis worldwide. There are two types of echinococcosis epidemics: cystic echinococcosis (CE) caused by the larvae of *Echinococcus granulosus* and alveolar echinococcosis (AE) caused by the larvae of *Echinococcus multilocularis* [1]. Echinococcosis is primarily found in pastoral or semi-agricultural-semi-pastoral areas in Western China, affecting 370 counties in provinces or autonomous regions such as Inner Mongolia, Yunnan, Tibet, Sichuan, Gansu, Qinghai, Ningxia, Xinjiang, and Shaanxi [2]. China echinococcosis endemic areas are separated into Qinghai-Tibet Plateau endemic areas and non-Qinghai-Tibet Plateau endemic areas based on geography and landform. There are 148 endemic counties on the Qinghai-Tibet Plateau, including all 74 counties in Tibet, 30 counties in Qinghai Province, 32 counties in Sichuan Province, 9 counties in Gansu Province, and 3 counties in Yunnan Province, while there are 222 endemic counties on the non-Qinghai-Tibet Plateau. The Qinghai-Tibet Plateau is the “hot spot” in terms of prevalence of human echinococcosis in China [2]. The epidemiological assessment of echinococcosis conducted from 2012 to 2016 reported the average detection rate of echinococcosis in the population of 1.28% in the Qinghai-Tibet Plateau and the non-Qinghai-Tibet Plateau with an average detection rate of 0.13% [3]. The detection rate in the Qinghai-Tibet Plateau is almost 10 times that in the non-Qinghai-Tibet Plateau because of animal husbandry and specific economy, culture, and production lifestyles [4]. Echinococcosis not only endangers people’s health and life but also stifles the healthy growth of agriculture and animal husbandry economies [5]. It is also one of the primary reasons why people in these epidemic areas “become poor and return to poverty” [6].

CE mostly damages and impairs the target organs of the liver, lungs, brain, spleen, kidneys, and heart, etc. [7]. CE’s liver cysts grow slowly, and population screening showed that more than half of the liver cysts did not change in size for 10 years. In addition, the early stages of CE and AE do not cause symptoms, and the lesions of CE and AE could remain asymptomatic for 10–15 years [8, 9]. AE, often known as “worm cancer,” is more dangerous than CE with a faster growth and death rate of up to 90% within 10 years if untreated or improperly treated [10, 11]. The cyst grows and ruptures as the disease advances, resulting in lethal anaphylactic shock. CE in China accounts for 40% of the disease burden worldwide, with as much as 398,000 disability-adjusted life years (DALYs) [12]. AE in China accounts for 90% of total cases and 91% of new cases worldwide [13].

In susceptible populations, echinococcosis can create major medical, social, and economic difficulties. The identification of susceptible populations is thus critical for prevention and control. Similarly, properly targeting health education to the most susceptible population will also help reduce or eliminate risk factors and improve the efficiency of echinococcosis prevention and control. It will also help optimize the allocation of public health resources. Most communities in the Qinghai-Tibet Plateau are Tibetans, and their lifestyles and customs differ from those of non-Qinghai-Tibet Plateau communities. Correlations were found between gender, age, education, occupation, and other societal traits and echinococcosis. However, most studies focused on only one population feature, and the other population characteristics were insufficiently detailed. One study used clustering analysis to identify vulnerable populations but only according to human demographics in Tibet Autonomous Region [14]. Thus, this work was undertaken as a comparative analysis of echinococcosis cases from Qinghai-Tibet Plateau and non-Qinghai-Tibet Plateau integrating together gender, age, education, occupation, and societal aspects.

## Materials and methods

### Source of data

Demographic information (gender, age, education, occupation, etc.) of echinococcosis patients, but also societal information (lifestyle, drinking water source, neighbors own dogs, feral dogs in the surrounding environment, and slaughter types) were collected from 2012 to 2016 in 370 endemic counties by cross-sectional cluster sampling. Human echinococcosis cases were identified using China's approved “Diagnostic criteria for echinococcosis” (WS 257–2006) by using portable B-mode ultrasonography, a standard in line with that of WHO [15]. Suspected cases were confirmed by two positive serological tests [15]. A questionnaire was used to collect the related life behavior factors of patients. Cases are standardized by gender, age, education, occupation, and other factors based on diagnosed cases (Table 1). Drinking water sources was classified according to the degree of cleanliness as level 1 (tap water and well water), level 2 (river water and spring water), and level 3 (water accumulation, ditch water, ponding).

**Table 1:** Population variables

Variables	Attributes
Gender	M (male), F (female)
Age	1 (< 21 years old), 2 (21–64), 3 (≥ 65 years old)
Education	Pre (preschool), I (illiteracy), P (primary), J (junior), S (senior), C (college and above)
Occupation	H (herdsman), HFa (semi-farmer and semi-herdsman), F (farmer), S (student), Pub (public officer), W (houseworker), M (monks and other religious figures), O (other)
Mode of residence	1 (settled), 2 (nomadism), 3 (winter settlement and summer nomadism), 4 (other)
Water quality	1 (drinking water cleanliness level 1), 2 (drinking water cleanliness level 2), 3 (drinking water cleanliness level 3)
Dogs in the surrounding environment	Yes, no
Neighbors own dogs	Yes, no

### Statistical analyses

Analyses were conducted using the SPSS 21.0 software package (IBM, Armonk, NY, USA). A chi-square test was used to compare the lifestyle composition of cases from Qinghai-Tibet Plateau and non-Qinghai-Tibet Plateau. Clustering analysis was used to group data objects as previously described [16]. The k-means algorithm was used to separate n objects into k clusters, each having a high similarity of data objects, whereas data objects inside the other clusters are less similar [17].

The procedure was the following [18]:

(i） Any k point was chosen as the initial cluster center.
(ii） The distance between the remaining points and the cluster center was calculated based on the minimum distance, as follows:
  $$dis(xi,xj)=\sqrt{{{\sum }_{d=1}^{D}({x}_{i,d} ,{x}_{j,d})}^{2}}$$
(iii） A new cluster center was calculated for each cluster.
(iv） Steps (ii) and (iii) were repeated until no new cluster centers could be created.

R 4.0.0 was used (R Development Core Team; R Foundation for Statistical Computing; Vienna, Austria) for k-means clustering.

## Results

### General situation

There were 4323 cases, including 1811 men and 2512 women, with a sex ratio of 1:1.39, accounting for 41.89% and 58.11% of cases, respectively. The youngest patient was 2 years old and the oldest 95 years old with a median age of 42. The Qinghai-Tibet Plateau was the host of 3286 cases while 1037 cases were found in the non-Qinghai-Tibet Plateau area (Table 2). CE represented 3194 cases, and AE was responsible for 1064 cases (Table 2). There were 65 cases of co-infections with CE and AE. In analysis CE cases comprised confirmed CE cases and co-infections with CE and AE. Similarly, AE cases included confirmed AE cases and co-infections with CE and AE.

**Table 2:** Echinococcosis cases in non-Qinghai-Tibet Plateau

Area	CE	AE	Co-infection of CE and AE	Total
Qinghai-tibet plateau	2210	1035	41	3286
Non-Qinghai-Tibet Plateau	984	29	24	1037
Total	3194	1064	65	4323

### Identification of common risk factors for both CE and AE

**Fig. 1:**
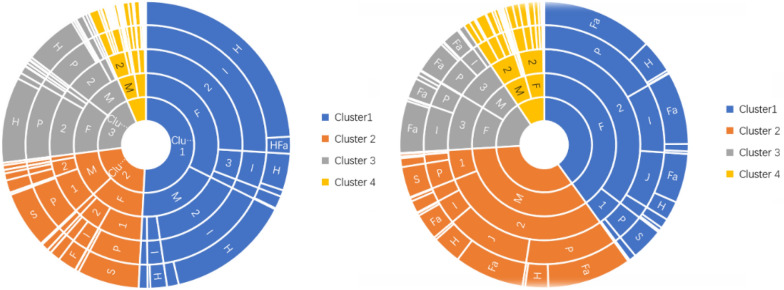
Cluster results of vulnerable populations in the Qinghai-Tibet Plateau and non-Qinghai-Tibet Plateau

The cluster analysis was performed on Qinghai-Tibet Plateau and non-Qinghai-Tibet Plateau cases, respectively. Each ring was built from the inside out based on proportion, gender, age, education, and occupation (Fig. 1).

### Cluster analysis of populations at risk in the Qinghai-Tibet Plateau

The range of k-clusters was defined as 2–7 [19, 20]. After determining the best k value, the instances from Qinghai-Tibet Plateau were grouped using *k* = 4 as the optimal cluster number (Fig. 1a). The first cluster comprised 1672 cases, essentially composed of women in age class 2, i.e. 21–64 years old, and illiterate herders (Fig. 1a). The second cluster comprised 730 cases, the majority being women under the age of 21 and with only primary school education (Fig. 1a). The third cluster was made of 675 individuals who were primarily women in age class 2 (21–64 years old), having a primary school education and working with herders. The last cluster comprised 209 individuals, the majority being men in age class 2 (21–64 years old) with a low educational level (primary school) and a religious profession.

### Cluster analysis of populations at risk in the non-Qinghai-Tibet Plateau

Four clusters were also found in the non-Qinghai-Tibet Plateau (Fig. 1b). The first one comprised 353 cases, mostly women and farmers of age class 2 (21–64 years) with primary school education. The second cluster grouped 296 individuals, mostly male farmers of age class 2 with elementary and junior middle school education. The 286 individuals making the third cluster were mostly elderly women in class age 3 (≥ 65 years old), uneducated and farmers. The last cluster included 102 cases comprised mostly men of age class 2 (21–64 years old), educated in junior high and senior high school, and having diverse occupational activities.

### Clustering according to lifestyle

#### Cluster analysis of CE cases

**Fig. 2:**
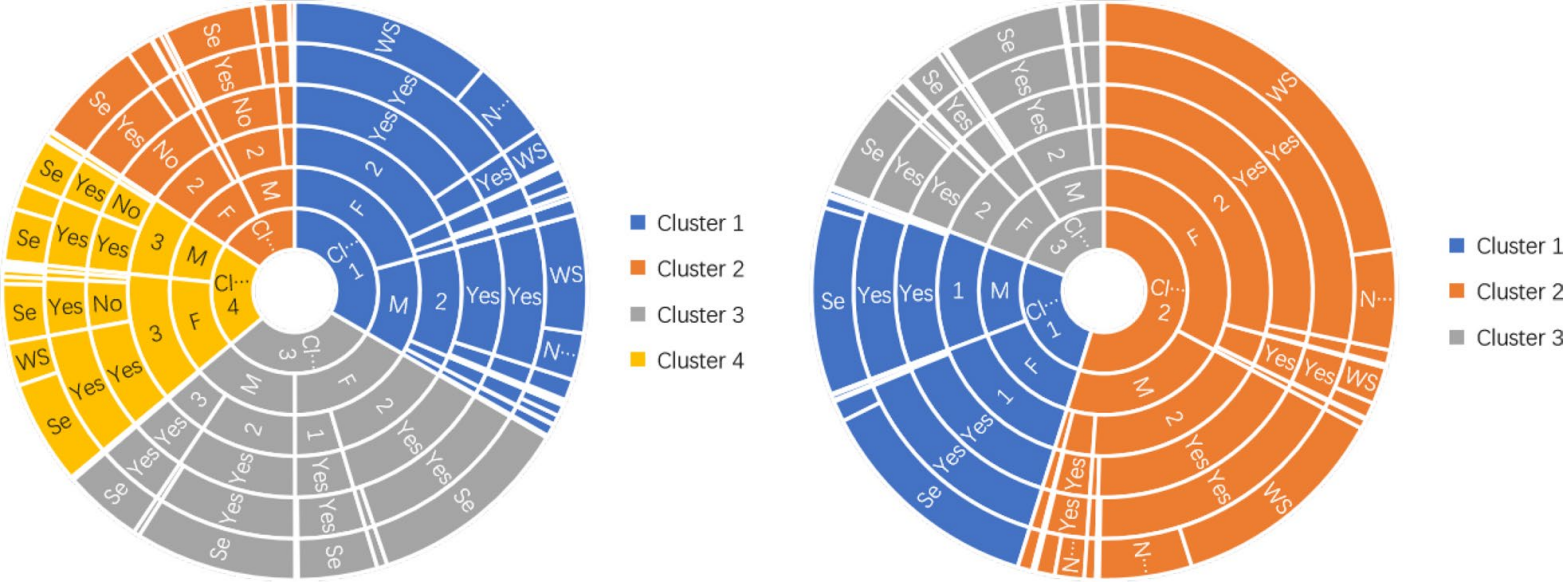
Clustering results for susceptible populations in CE and AE cases

There were 3259 CE cases (including co-infection of CE and AE). Four clusters were found (Fig. 2a). The first comprised 1062 individuals, mostly women in age class 2 (21–64), living in a winter settlement and summer nomadic lifestyle, with dogs in the vicinity and feral dogs in surrounding areas. The second cluster grouped 506 cases, also mostly women in age class 2 (21–64), living in a permanent residence, without dogs in the vicinity but with feral dogs in surrounding areas. The third cluster gathered 982 individuals and corresponded to women in age class 2, living in permanent settlements with dogs in the vicinity and feral dogs in the surrounding environment. The last cluster comprised 709 individuals, mostly women again but from age class 3 (≥ 65 years old), also living in permanent settlements, with both domestic dogs in the vicinity and feral dogs in the surrounding environment.

#### Cluster analysis AE cases

There were 1129 AE cases (including co-infection of CE and AE). Only three clusters were identified in the case of AE (Fig. 2b). The first cluster comprised 290 cases, mostly women in age class 1 (< 21 years old), living in permanent settlements with dogs in the vicinity and feral dogs in the surrounding environment. The second cluster grouped 608 individuals, also mostly women but in age class 2 (21–64 years old) with a lifestyle of permanent winter residence and summer nomadism and with domestic dogs in the vicinity and feral dogs in the surrounding environment. The last cluster grouped 231 individuals and predominantly comprised men in age class 2 (21–64 years old) in permanent settlements and with domestic dogs in the vicinity and feral dogs in the surrounding area.

### Lifestyle and human echinococcosis

The lifestyle patterns of human echinococcosis cases of Qinghai-Tibet Plateau and non-Qinghai-Tibet Plateau were analyzed using the following parameters: living patterns (permanent or nomadic), drinking water sources, neighborhood domestic dogs situation, and presence of feral dogs in the environment.

#### Lifestyle clustering analysis of Qinghai-Tibet Plateau cases

**Fig. 3:**
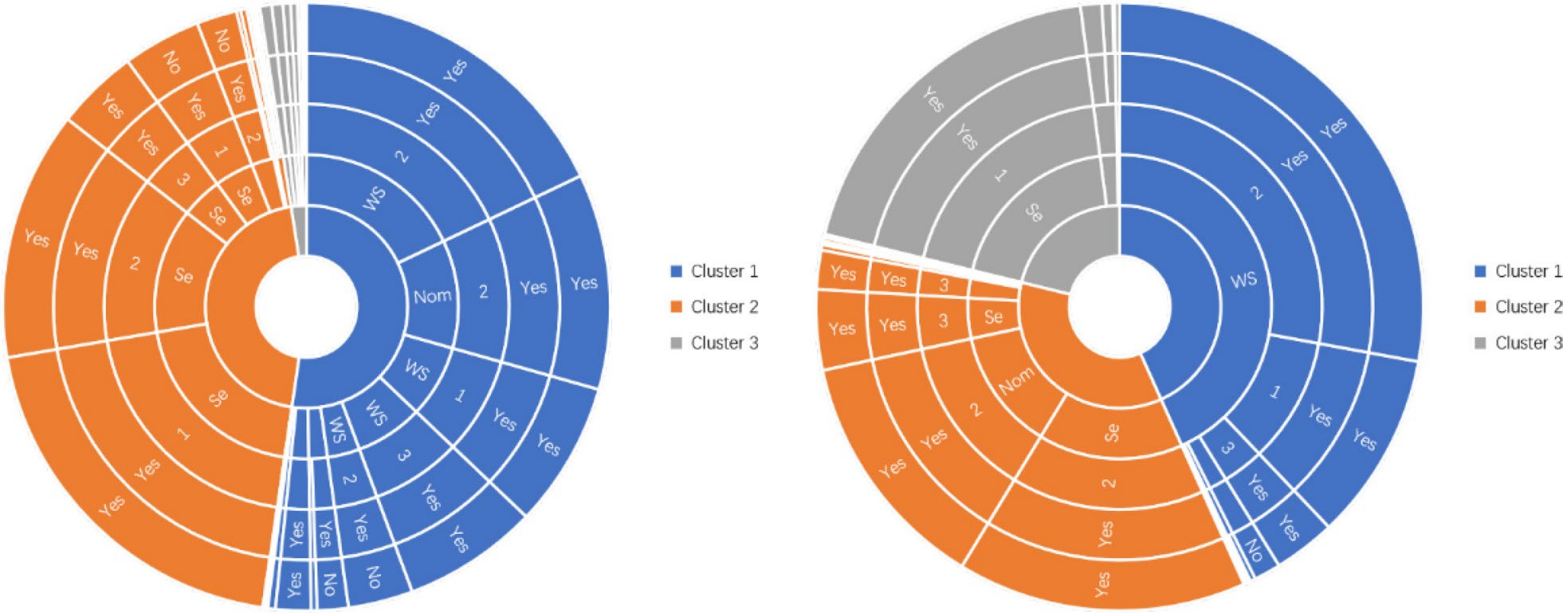
Results of lifestyle clustering in cases for CE and AE in Qinghai-Tibet Plateau

There were 2251 CE cases in Qinghai-Tibet Plateau (including co-infection of CE and AE). We used *k* = 3 as the best clustering number to cluster CE cases in Qinghai-Tibet Plateau (Fig. 3). There were 1152 cases in the first category, concentrated in groups whose lifestyle was winter residence and summer nomadism, drinking water cleanliness was level 2, neighbors had dogs, and there were feral dogs in the surrounding area. The settlement lifestyle, level 2 and 3 drinking water cleanliness, and domestic and feral dogs in the surrounding environment were factors in the 997 cases in the second category. There were 102 cases in the third category, mainly concentrated in groups where the living style was settlement, there was drinking water level 1 cleanliness, neighbors had no domestic dogs, and the surrounding environment had no feral dogs. For AE, there were 1076 AE cases in Qinghai-Tibet Plateau (including co-infection of CE and AE). There were 458 cases in the first category, concentrated in groups whose lifestyle was winter residence and summer nomadism, drinking water cleanliness was level 2, and neighbors had dogs and feral dogs in the surrounding area. Nomadic lifestyle, level 2 and 3 drinking water cleanliness, and domestic and feral dogs present in the surrounding environment were factors in the 376 cases in the second category. There were 242 cases in the third category, concentrated in groups where the lifestyle was: settlement, level 1 drinking water cleanliness, neighbors having dogs, and feral dogs in the surrounding environment.

#### Lifestyle clustering analysis of non-Qinghai-Tibet Plateau cases

**Fig. 4:**
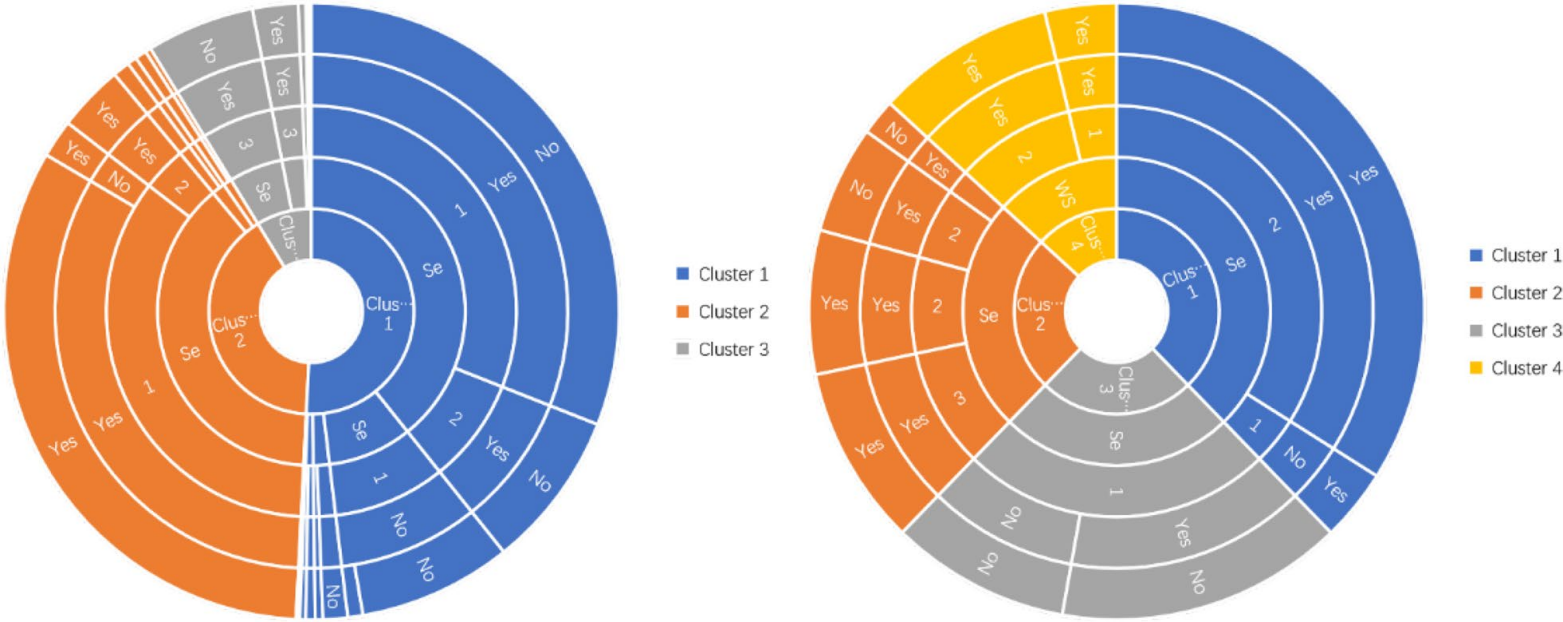
Results of lifestyle clustering in cases of CE and AE in non-Qinghai-Tibet Plateau

There were 1008 CE cases in non-Qinghai-Tibet Plateau (including co-infection of CE and AE). The findings of clustering cases for CE in non-Qinghai-Tibet Plateau with *k* = 3 as the optimal clustering number are displayed in Fig. 4. In the first group, there were 510 cases, concentrated in the population who had settlement lifestyle, level 1 cleanliness of drinking water, neighbors with dogs, and no feral dogs in the surrounding environment. In the second category, there were 406 cases, concentrated in groups with a settled lifestyle, level 1 cleanliness of drinking water, neighbors with dogs, and feral dogs in the surrounding environment. There were 92 cases in the third group, concentrated in groups with a settled lifestyle, level 3 cleanliness of drinking water, neighbors with dogs, and no feral dogs in the surrounding environment. For AE, there were 53 AE cases in non-Qinghai-Tibet Plateau (including co-infection of CE and AE). In the first group there were 20 cases, concentrated in the population with settled lifestyle, level 2 cleanliness of drinking water, neighbors with dogs, and feral dogs in the surrounding environment. In the second category, there were 13 cases, concentrated in groups with a settled lifestyle, level 3 cleanliness of drinking water, neighbors with dogs, and feral dogs in the surrounding environment. There were 13 cases in the third group, concentrated in groups with a settled lifestyle, level 1 cleanliness of drinking water, neighbors with dogs, and no feral dogs in the surrounding environment. Seven cases in the fourth category were primarily concentrated in the population whose lifestyle consisted of winter residence and summer nomadism, the purity of drinking water was secondary water, neighbors owned dogs, and feral dogs in the surrounding environment.

#### Comparison of behavior patterns in Qinghai-Tibet Plateau and non-Qinghai-Tibet Plateau cases

Summer nomadism and winter permanent settlements were more frequent in the Qinghai-Tibet plateau cases than in non-Qinghai-Tibet Plateau ones. The proportion of cleanliness levels 2 and 3 of drinking water source selection was higher in Qinghai-Tibet plateau cases than in the non-Qinghai-Tibet Plateau ones; for the latter, the proportion of cleanliness level 1 was higher. As indicated in Table 3, the presence of domestic dogs in the neighborhood and that of feral dogs in the surrounding environment were higher on the Qinghai-Tibet Plateau than the non-Qinghai-Tibet Plateau. In Qinghai-Tibet Plateau, the families of the 1202 cases (36.6%) did not choose to slaughter livestock. Among the families, a total of 1101 (33.5%) cases chose to slaughter livestock at home. Only the families of 146 cases (4.4%) chose to slaughter animals in centralized facilities, while the families of 837 (25.5%) cases used sporadic random livestock slaughter. A total of 276 non-Qinghai-Tibet Plateau subjects (26.6%) did not slaughter livestock. As stated in Table 3, the families of 551 subjects (53.1%) chose to slaughter livestock at home, and the families of 133 (12.8%) cases were involved in intensive slaughter.

**Table 3:** Comparison of the lifestyle of cases in Qinghai-Tibet Plateau and non-Qinghai-Tibet Plateau cases

	Cases in Qinghai-Tibet Plateau	Non-Qinghai-Tibet Plateau cases	*χ*^2^	*P*
Mode of living				
Settlement	1478 (45.0%)	981 (94.6%)	797.72	*P* < *0.05*
Nomadism	1328 (40.4%)	34 (3.3%)		
Winter settlement and summer nomadism	480 (14.6%)	21 (2.0%)		
Other	0 (0.0%)	1 (0.1%)		
Sources of drinking water				
Level 1 cleanliness	1123 (34.2%)	783 (75.5%)	565.55	*P* < *0.05*
Level 2 cleanliness	1726 (52.5%)	160 (15.4%)		
Level 3 cleanliness	437 (13.3%)	94 (9.4%)		
Neighbor own dogs				
Yes	3189 (97.0%)	922 (88.9%)	111.93	*P* < *0.05*
No	97 (3.0%)	115 (11.1%)		
Feral dogs in surrounding environment				
Yes	2943 (89.6%)	455 (43.9%)	978.18	*P* < *0.05*
No	343 (21.4%)	582 (56.1%)		
Slaughter types				
Concentrated slaughtering	146 (4.4%)	133 (12.8%)	309.60	*P* < *0.05*
Family slaughtering	1101 (33.5%)	551 (53.1%)		
Sporadic slaughtering	837 (25.5%)	77 (7.4%)		
No slaughtering	1202 (36.6%)	276 (26.6%)		

## Discussion

The specific characteristic of the echinococcosis epidemic in China is that dogs are the common source of infection for both CE and AE. CE and AE display common risk factors with 115 counties endemic for both diseases. The natural and geographical environment, climate, and customs of the Qinghai-Tibet Plateau are different from those of the non-Qinghai-Tibet Plateau, as are the transmission cycles of *E. granulosus* and *E. multilocularis*. Population characteristics and risk factors in the Qinghai-Tibet Plateau and in the non-Qinghai-Tibet Plateau regions are thus also different. Some risk factors are similar in both regions, such as middle-aged populations, low education level, and occupations as herdsman or farmers [14]. Within the Qinghai-Tibet Plateau some groups at risk of CE and AE are similar: middle-aged, low-educated women who spend most of their time at home [21]. They frequently feed dogs and livestock, collect cow dung for fuel, shear wool, and perform other household duties. Therefore, they are extremely susceptible to *E. granulosus* and *E. multilocularis* [21] infection. Age and low education level are risk factors for echinococcosis based on community studies conducted in Argentina [22]. According to an epidemiological survey conducted in Chile, poor socioeconomic status and lack of education have also been linked to CE in humans [23].

In both the Qinghai-Tibet Plateau and the non-Qinghai-Plateau, the susceptibility factors for echinococcosis are centered on children. However, in the former, children accounted for 15%, while in the latter 6.94%. Because of the pastoral lifestyle and conditions, children are expected to help with household duties from a young age. The process involves contact with cattle and possible direct or indirect contact with dogs, which is exacerbated by inadequate local sanitation and lack of hygiene awareness among children. There is thus a considerable chance of contracting echinococcosis. Unlike schistosomiasis, malaria, and other parasitic infections, echinococcosis has a slow onset and hidden course. Most patients have been infected as children and are very vulnerable since the symptoms are unclear and difficult to detect and treat [24].

Therefore, improving surveillance, prevention, and control of echinococcosis infection in children is critical. It is possible to carry out serological and ultrasonic imaging examinations for echinococcosis as well as health education programs in schools on a regular basis. It is crucial to expand health education and raise public awareness of echinococcosis prevention and control. Religious practices, in particular when associated with poor education, might be an aggravating factor in the Qinghai-Tibet Plateau. People in this region are Buddhists and thus praise and highly respect all life forms. Lamas in temples will often take in or feed abandoned stray dogs, leading to a higher concentration of these dogs, which represent a major risk of infection near temples [20, 21].

The drinking water source is also a major risk factor [25]. *Echinococcus granulosus* eggs can only be inactivated after being stored at −80 °C for at least 7 days, and they can survive for > 200 days at 7 °C and 50 days at 21 °C [26]. *Echinococcus multilocularis* eggs can also survive in water at 4 °C for 16 months [27]. Parasitic eggs are very resistant in the environment and can even withstand common disinfectants. Boiling is the most effective method to inactivate *Echinococcus* eggs [21]. Therefore, drinking water is the most common path of echinococcosis infection [28]. Springs, rivers, ponds, and ditches, but also wells and even tap water, can be polluted by *Echinococcus* eggs in dog feces. The traditional Tibetan lifestyle of many people in the Qinghai-Tibet Plateau is to settle in the winter in permanent residences and to follow a nomadic lifestyle in summer. During this nomadic period, people and dogs share the same water supply, which is not boiled, tap or well water as well as surface water, such as springs and rivers, which are often of poor quality. *Echinococcus* eggs in dog feces are likely to pollute surface water. Indeed, nomadic herders often prefer pastures with natural water sources to set up tents, and livestock and dog excrement is randomly discharged in the area, polluting the surrounding water sources [29]. Drinking or using contaminated surface water increases the risk of infection. Furthermore, herdsmen traditionally collect cow dungs as fuel, which will be brought into tents for usage [30]. These customs also increase the risk of infection with echinococcosis. Of course, drinking clean water minimizes the risk of infection, and thus tap water is an echinococcosis protective factor [21, 31, 32]. However, it is not compatible with the traditional semi-nomadic way of life.

Although most patients in the non-Qinghai-Tibet Plateau tend to drink cleaner water, economic development in some places is lagging. Water storage is still part of a traditional lifestyle, so water purity cannot be ensured. However, building water pipelines in CE- and AE-endemic areas is challenging because of geographical and climatic factors, particularly on the Qinghai-Tibet Plateau. Depending on local economic and environmental conditions, appropriate actions should be taken to disinfect and treat local household water, improve water source management, and install disinfection or filtration units to enable local populations to access safe domestic water as well as limit the danger of exposure to *Echinococcus* eggs.

On Qinghai-Tibet Plateau, the presence of many both domestic and feral dogs greatly increases the risk of contamination, in particular for children. Their health awareness is low, and they lack prevention expertise. As aggravating factors, geographical characteristics in endemic areas, adverse climatic conditions, and economic factors make access to clean drinking water problematic, thus increasing the likelihood of infection in children. Most studies revealed the presence of both feral dogs and domestic dogs, particularly on the Qinghai-Tibet Plateau. However, feral dogs are less common in non-Tibetan societies, probably because of different cultural backgrounds [33]. Canines are the ultimate hosts of *Echinococcus*, and their faeces contain *Echinococcus* eggs and can contaminate the environment and water sources, as does animal hair [34]. A survey of domestic dogs in an epidemic area showed the presence of positive *Echinococcus* antigens in 4.25% of dog feces [5]. Feral dogs are also prevalent in CE- and AE-endemic areas [35, 36]. Dog management and control are critical measures in preventing and controlling echinococcosis as the zoonotic risk of CE and AE increases with the number of dogs and time dogs are maintained [21]. Tibet Autonomous Region has implemented comprehensive prevention and control measures focusing on controlling the infectious source of echinococcosis and achieved good results [37]. The Tibetan semi-nomadic way of life is unique and should be maintained. However, it should include efficient control measures related to animal husbandry and dogs; otherwise, echinococcosis will remain a severe human concern [30]. However, these control measures are difficult to implement.

The main source of income in the agricultural and pastoral areas is livestock raising, which uses dogs to look after and protect herds. Cattle and sheep are also intermediate hosts of *E. granulosus*. During domestic and sporadic slaughtering, the internal organs of livestock are generally fed to dogs along with the parasites they may contain [36]. In-house slaughter represents a 4.67 times higher risk of echinococcosis [38]. Centralized slaughter can successfully cut off this transmission pathway, reducing the risk of dog illness and thus safeguarding the health of residents. The first strategy for preventing and controlling echinococcosis is to decrease or destroy *Echinococcus* eggs, and the second is to reduce the probability of interaction with *Echinococcus* eggs [39]. Human proximity to definitive hosts (dogs) and animal intermediate hosts (livestock or small mammals) will increases the potential risk of echinococcosis (CE and AE) [30].

Non-Qinghai-Tibet Plateau is dominated by CE prevalence. The lifestyle of local residents is different from that of residents in the Qinghai-Tibet Plateau, being mainly settled. So they have access to clean tap drinking water. In this case, the main risk factor for CE is the presence of domestic and feral dogs. Because the geographical and natural environment is different from that of the Qinghai-Tibet Plateau, the main risk factors for AE are dogs in the surrounding environment and unclean drinking water.

Canine deworming and health education, particularly for vulnerable communities, are essential to enhance their understanding of the mechanisms of infection and improve inadequate hygiene practices. This study does have some drawbacks. The collection of influencing factors was limited, such as for CE, the infection rate of livestock, and for AE, the infection rate of small rodents.

## Conclusion

In this study, the k-means clustering analysis revealed that the populations susceptible to echinococcosis were mostly middle-aged, poorly educated semi-nomadic herdsmen. However, on Qinghai-Tibet Plateau a specific group of patients was young pupils. This is a serious signal that special attention must be paid to this group. We should increase their health education and raise their awareness about avoiding and controlling echinococcosis. Adapting some traditions and lifestyles might be important to reduce the risk of infection. Simultaneously, in light of risk factors such as feral dogs, dog breeding, polluted drinking water, and domestic animal slaughter practices, we should increase management measures, improve relevant health regulations, improve sanitation conditions, and provide adequate technology for water purification, centralized slaughter, monitoring, and deworming.

## Data Availability

The original contributions presented in the study are included in the article material; further inquiries can be directed to the corresponding author.

## Abbreviations

AE: Alveolar echinococcosis
CE: Cystic echinococcosis
DALYs: Disability-adjusted life years
